# Anticoagulants: A Review of the Pharmacology, Dosing, and Complications

**DOI:** 10.1007/s40138-013-0014-6

**Published:** 2013-04-21

**Authors:** Mohammed Alquwaizani, Leo Buckley, Christopher Adams, John Fanikos

**Affiliations:** Pharmacy Department, Brigham and Women’s Hospital, 75 Francis Street, Boston, MA 02115 USA

**Keywords:** Thrombosis, Coagulation, Anticoagulants, Unfractionated heparin, Low molecular weight heparins, Direct thrombin inhibitors, Warfarin, Dabigatran, Rivaroxaban, Apixaban

## Abstract

Anticoagulants remain the primary strategy for the prevention and treatment of thrombosis. Unfractionated heparin, low molecular weight heparin, fondaparinux, and warfarin have been studied and employed extensively with direct thrombin inhibitors typically reserved for patients with complications or those requiring intervention. Novel oral anticoagulants have emerged from clinical development and are expected to replace older agents with their ease of use and more favorable pharmacodynamic profiles. Hemorrhage is the main concerning adverse event with all anticoagulants. With their ubiquitous use, it becomes important for clinicians to have a sound understanding of anticoagulant pharmacology, dosing, and toxicity.

## Introduction

Anticoagulants are the cornerstone therapy for thrombosis prevention and treatment. While anticoagulants are commonly employed, their use is often associated with adverse drug events and increased readmission rates. In older patients presenting to an Emergency Department with a warfarin adverse drug event, about half required hospitalization [1]. Despite novel anticoagulants being touted as replacements for warfarin and heparin products, rivaroxaban has been associated with serious thrombotic events while dabigatran has been associated with serious bleeding [2, 3]. Since anticoagulant use enhances the risk for Emergency Department visits by as much as 35-fold [4], clinicians must be familiar with anticoagulants, their pharmacological properties, pharmacodynamics, dosing, monitoring, and toxicity.

## Pathophysiology

**Fig. 1:**
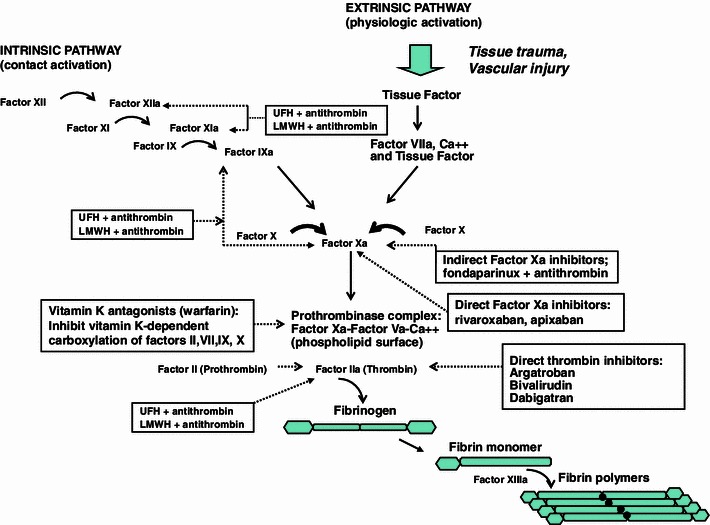
The coagulation cascade is comprised of the intrinsic (contact activation) pathway and the extrinsic (tissue factor) pathway. Each pathway generates a series of reactions in which inactive circulating enzymes and their co-factors are activated. These activated factors then catalyze the next reactions in the cascade. Thrombin plays a pivotal role by triggering the conversion of soluble fibrinogen to insoluble fibrin monomers, which serve as the foundation for thrombus formation. Thrombin also activates factors VIII, V, and XIII. Factor XIII generates the covalent bonds that link fibrin strands ensuring structural integrity. Anticoagulants, either through their interaction with Antithrombin (AT) or through a direct inhibition of thrombin, interrupt these enzymatic reactions

The coagulation cascade is triggered by tissue factor release from tissue trauma or vascular injury (Fig. 1) [5]. Tissue factor forms a complex with factor VIIa in the presence of calcium and cleaves clotting factors X and IX to their activated forms (factors Xa and IXa). The prothrombinase complex is then assembled on a phospholipid membrane and cleaves prothrombin (factor II) to factor IIa (thrombin). Thrombin is one of the most potent activators of primary (platelet-mediated) and secondary (clotting factor-mediated) hemostasis. Thrombin may also potentiate clot formation by fibrin polymerization, platelet receptor activation, endothelium activation, and activation of factors V, VIII, XI, and XIII. Anticoagulant agents can inhibit thrombogenesis by altering various pathways within the clotting cascade or by targeting thrombin directly, attenuating thrombin generation. Indirect inhibitors, however, target and bind to naturally occurring plasma cofactors, such as antithrombin (AT), catalyzing their interaction with clotting enzymes [5].

## Pharmacology of Heparins and Fondaparinux

Unfractionated heparin (UFH) and low molecular weight heparin (LMWH) are the anticoagulants of choice in acute thrombosis due to their rapid onset of antithrombotic activity. Since heparins are dependent on the presence of AT for clotting factor inhibition, they are considered indirect anticoagulants (Table 1) [6••, 7, 8]. Heparins have no fibrinolytic activity and will not lyse existing thrombi. Heparins contain an active pentasaccharide sequence that binds to AT. Once heparin binds and activates AT, it can readily dissociate and bind to additional AT, providing a continuous anticoagulant effect. This binding produces a conformational change, accelerating AT binding and inactivation of coagulation factors XIIa, IXa, XIa, Xa and thrombin. The active pentasaccharide sequence responsible for catalyzing AT is found on one-third and one-fifth of the chains of UFH and LMWH, respectively. Fondaparinux is a synthetic analog of the naturally occurring pentasaccharide found in heparins [6••, 7, 8]. Fondaparinux selectively and irreversibly binds to AT. This results in neutralization of factor Xa, which ultimately inhibits thrombin formation and thrombus development.

**Table 1:** Comparison of the pharmacologic features of heparin and its derivatives

Features	Heparin	LMWH	Fondaparinux
Source	Biological	Biological	Synthetic
Molecular weight (Da)	15,000	5,000	1,500
Target	Xa:IIa	Xa > IIa	Xa
Bioavailability (%)	30	90	100
Half-life (h)	1	4	17
Renal excretion	No	Yes	Yes
Protamine reversal	Complete	Partial	None
Incidence of HIT (%)	<5.0	<1.0	Case reports

*Da* Dalton, *h* hours, *HIT* heparin-induced thrombocytopenia

## Unfractionated Heparin

### Pharmacodynamics and Monitoring

Intravenous (IV) infusion or subcutaneous injections are the available routes for UFH administration and IV is preferred [6••, 7–9]. When given via subcutaneous injection for therapeutic anticoagulation, doses need to be large enough (>30,000 U/day) to overcome UFHs low bioavailability. UFH readily binds to plasma proteins, which contributes to its variable anticoagulant response after parenteral administration. Despite these limitations IV administration rapidly achieves therapeutic plasma concentrations that can be effectively monitored and adjusted based on infusion rates [7].

UFH clearance from the systemic circulation is dose-related and occurs through two independent mechanisms [6••, 8]. The initial phase is the rapid and saturable binding to endothelial cells, macrophages, and local proteins where UFH is depolymerized. The second phase is a slower, non-saturable, renal-mediated clearance. At therapeutic doses, UFH is cleared primarily via depolymerization, with the higher molecular weight chains being cleared more rapidly than lower weight counterparts. As clearance becomes dependant on the kidney, increased or prolonged UFH dosing provides a disproportionate increase in both the intensity and the duration of the anticoagulant effect.

The anticoagulant response to UFH administration is monitored using the activated partial thromboplastin time (aPTT). The aPTT should be measured every 6 h with IV administration, and doses adjusted accordingly, until the patient has sustainable therapeutic levels. Once steady state is reached the frequency of monitoring can be extended [8, 10].

To overcome variables delivering UFH, weight-based dosing nomograms are recommended for treatment of thromboembolic disease. Dosing nomograms have been associated with significantly higher initial UFH doses, shorter time to therapeutic activated aPTT, and no increase in bleeding events. UFH dosing nomograms will differ from hospital to hospital due to differences in thromboplastin agents and inter-laboratory standardizations in aPTT measurements [10].

### Clinical Indications

Clinical indications for UFH include treatment of acute coronary syndromes (ACS), treatment or prevention of venous thromboembolism (VTE), bridge therapy for atrial fibrillation (AF), and cardioversion (Table 2) [6••, 11–13]. UFH utilization has diminished with LMWH and fondaparinux availability and their superior pharmacokinetic profiles [6••, 7]. UFH, with a short half-life and reversal capability, remains the best option in patients requiring higher UFH doses, in patients with underlying bleeding risk, or in those critically ill with organ dysfunction. Patients with fluctuating renal function or with a creatinine clearance less than 30 mL/min are not candidates for LMWH or fondaparinux due to the risk of accumulation and increased bleeding risk [14]. When used for thromboprophylaxis in medical patients, three times daily UFH dosing provides better efficacy in preventing VTE events compared to twice daily dosing but generates more major bleeding episodes [15].

**Table 2:** Clinical uses of UFH

Drugs	Indications	Dosing, timing, duration	Monitoring	Precautions
Unfractionated heparin	Treatment of VTE	80 U/kg bolus then 18 U/kg/h infusion adjusted to maintain aPTT 2–2.5 times control or per local heparin nomogram	aPTT: at least 6 h after initiation, then at least once daily Anti-Xa Levels (alternative if available, consider if patients with heparin resistance) CBC HIT antibody testing (not warranted in the absence of thrombocytopenia, thrombosis, heparin-induced skin lesions, or other signs pointing to a potential diagnosis of HIT Signs and symptoms of bleeding	Allergic or hypersensitivity-type reactions Congenital or acquired bleeding disorders Indwelling epidural catheter Gastrointestinal ulceration and ongoing tube drainage of the small intestine or stomach Hepatic disease with impaired hemostasis Hereditary AT III deficiency and concurrent use of AT Menstruation Neonates and infants weighing <10 kg Premature infants weighing less than 1 kg Risk of delayed onset of HIT and HITT
	Treatment of ACS	IV bolus: 60 U/kg (max 4,000 U) 12 U/kg/h (max 1,000 U) ± fibrinolysis, adjusted to maintain aPTT 1.5–2 times control or per local heparin nomogram		
	Bridge therapy for AF, cardioversion	IV infusion: 60–80 U/kg bolus Target aPTT, 60 s, range 50–70 s		
	Prophylaxis of VTE in the medically ill or surgical population	5,000 U SC every 8–12 h		
	Prophylaxis of VTE in pregnancy (with prior VTE)	7,500–15,000 U SC every 12 h		

*VTE* venous thromboembolism, *aPTT* activated partial thromboplastin time, *CBC* complete blood count, *HIT* heparin-induced thrombocytopenia, *HITT* heparin-induced thrombocytopenia and thrombosis, *ACS* acute coronary syndrome, *IV* intravenous, *SC* subcutaneous

### Complications and Reversal of Effect

The major complications of UFH therapy include bleeding (major bleeding, 0–7 %; fatal bleeding, 0–3 %) and heparin-induced thrombocytopenia (HIT, 1–5 %). Patients receiving UFH for periods of more than 1 month are also at an increased risk for osteoporosis and development of vertebral fractures (approximately 2 % incidence) [16]. Hemorrhagic episodes are associated with the intensity of anticoagulation, route of administration (continuous infusions are associated with lower rates), and concomitant use of glycoprotein (gp) IIB/IIIA inhibitors, aspirin or fibrinolytic therapy [16–18]. The relationship between supratherapeutic levels of UFH (elevated aPTT, heparin levels or anti-Xa levels) and major bleeding is not well established and has not been prospectively compared in clinical trials. Major bleeding can occur within therapeutic levels of anticoagulation. Patient-specific risk factors are the most important consideration when determining the bleeding risk, including: age, gender, renal failure, low body weight, and excessive alcohol consumption [16–18].

Anticoagulation management before and after surgery is a patient specific, risk versus benefit decision. It is based on the procedure and patient’s risk factors for bleeding and thrombosis. For patients requiring peri-operative anticoagulation in elective procedures or surgery, discontinuing therapeutic IV UFH doses 4 h prior to the procedure and measuring an aPTT is usually sufficient, as normal hemostasis is restored in this time frame in most cases. If the aPTT remains elevated, then hourly measurements are advised until the aPTT returns to baseline [19–21]. Therapeutic UFH therapy can be restarted 12 h after major surgery, but should be delayed longer for evidence of continued bleeding. In patients receiving low-dose UFH subcutaneously, there is no contraindication to neuraxial techniques, as the risk for developing spinal hematoma appears to be minimal. In patients who are to receive intraoperative anticoagulation with UFH, the UFH infusion should be started at least 1 h after needle placement. Indwelling catheters should be removed 2–4 h after discontinuation of the UFH infusion and only after the patient’s coagulation status has been assessed [22].

Since UFH has a short half-life, reversal is not required in most bleed episodes. The treatment of clinically severe UFH-related bleeding includes anti-heparin therapy (protamine sulfate), transfusion therapy, and supportive care. Protamine dosing is dependent on timing of the last UFH dose. For immediate reversal (<30 min since the last UFH dose), 1 mg of protamine is administered for every 100 U of UFH and a follow up aPTT can evaluate the reversal response. When UFH is given as a continuous IV infusion, only UFH delivered during the preceding 2–2.5 h should be included in the calculation to determine the protamine dose. If the UFH dose is unknown, protamine 50 mg can be administered slowly over 10 min followed by serial measurements of aPTT. Severe adverse reactions to protamine, such as hypotension and bradycardia, are common. Reaction severity may be reduced by slowing the administration over 1–3 min (maximum administration rate is 5 mg/min). Allergic responses to protamine are more common in patients who have been previously exposed to the drug for UFH neutralization, or treated with protamine-containing insulin (neutral protamine Hagedorn insulin), have undergone vasectomy, or have hypersensitivity to fish. Patients at risk of developing anti-protamine antibodies can be pretreated with corticosteroid and anti-histamine medications [23–24].

## Low Molecular Weight Heparins

### Pharmacodynamics and Monitoring

LMWHs have increased bioavailability after subcutaneous injection, renal clearance that is dose-independent, and a longer half-life (17–21 h) when compared to UFH. LMWHs are administered in fixed doses for thromboprophylaxis, or in total body weight adjusted doses for therapeutic anticoagulation (Table 3) [5, 25].

**Table 3:** Clinical uses of LMWHs

Drugs	Indications	Dosing, timing, duration	Monitoring	Precautions
Enoxaparin (Lovenox™)	Treatment of VTE [2]	1 mg/kg SC every 12 h OR 1.5 mg/kg SC every 24 h	Anti-Xa level in with significant renal impairment, those experiencing bleeding or abnormal coagulation parameters, pregnant patients, obese or low-weight patients, and children CBC Serum creatinine HIT antibody testing (not warranted in the absence of thrombocytopenia, thrombosis, heparin-induced skin lesions, or other signs pointing to a potential diagnosis of HIT Signs and symptoms of bleeding	Indwelling epidural catheter Recent spinal or ophthalmologic surgery History of recent major bleed (gastrointestinal, intracranial, etc.) Congenital or acquired bleeding disorders Bacterial endocarditis History of heparin-induced thrombocytopenia Liver disease Renal impairment (CrCl <30 mL/min), consider UFH Concomitant use of antithrombotic drugs Diabetic retinopathy Uncontrolled hypertension
		CrCl <30 mL/min: 1 mg/kg SC every 24 h		
	Treatment of ACS [7]	ST-segment elevation MI: 30 mg bolus IV plus 1 mg/kg with tenecteplase followed by 1 mg/kg SC every 12 h Unstable angina/non-Q wave MI: 1 mg/kg SC every 12 h		
		CrCl <30 mL/min: not recommended		
	Prophylaxis/bridge therapy for AF/cardioversion [3]	1 mg/kg SC every 12 h OR 1.5 mg/kg SC every 24 h		
		CrCl <30 mL/min: 1 mg/kg SC every 24 h		
	Prophylaxis of VTE in the medically ill or surgical population	40 mg SC every 24 h		
		CrCl <30 mL/min: 1 mg/kg SC daily		
	Prophylaxis of VTE in the trauma patients	30 mg SC every 12 h OR 40 mg SC every 24 h		
Dalteparin (Fragmin™)	Treatment of VTE	<56 kg: 10,000 IU SC daily 57–68 kg: 12,500 IU SC daily 83–98 kg: 18,000 IU SC daily >99 kg: 18,000 IU SC daily		
	Treatment of ACS	120 IU/kg SC every 12 h (MAX 10,000 IU/dose)		
	Prophylaxis of VTE after hip or other major surgery (first month) [8]	Initial dose: 2,500 IU SC once Maintenance: 2,500–5,000 IU SC every 24 h [8]		
	Prophylaxis of VTE in the medically ill or surgical population	5,000 IU SC every 24 h		
Tinzaparin (Innohep™)	Treatment of DVT	175 international units anti-Xa/kg SC daily		

*VTE* venous thromboembolism, *SC* subcutaneous, *h* hours, *CrCl* creatinine clearance using the Cockroft–Gault Equation, *CBC* complete blood count, *HIT* heparin-induced thrombocytopenia, *ACS* acute coronary syndrome, *IV* intravenous, *IU* international units

With their predictable dose response (peak anti-Xa activity occurring 3–5 h after injection) laboratory monitoring is usually not necessary. Anti-Xa monitoring is an option in high-risk patient populations (renal insufficiency, obesity, pregnancy, non-compliance) where dosing adjustments may be required to tailor therapy. In these cases anti-Xa plasma levels are drawn 4 h after administration, and subsequent dosing adjusted to peak target levels of 0.5–1.1 IU/mL [26, 27]. Anti-Xa tests should be monitored and interpreted per the manufacturer of the specific LMWH being used.

### Clinical Indications

For medically ill and post-operative patients requiring parenteral VTE prophylaxis, LMWHs have become a suitable replacement for UFH [28, 29]. LMWHs require fewer injections and produce fewer adverse events. In hospitalized medical patients receiving thromboprophylaxis, LMWH was associated with a lower risk of deep vein thrombosis (DVT), fewer injection site hematomas, and no differences in bleeding when compared with UFH [30]. LMWHs have largely replaced IV UFH in patients with acute VTE who are able to continue therapy, unmonitored in the ambulatory setting [31]. In ACS, patients with ST-segment elevation myocardial infarction treated with fibrinolysis and LMWH had a lower incidence of death or non-fatal recurrent myocardial infarction but a higher rate of major bleeding than those treated with fibrinolysis and UFH [32]. Similarly, in unstable angina/non-ST-segment elevation myocardial infarction, LMWH therapy reduced the incidence of death, myocardial infarction, or urgent revascularization when compared to UFH [33].

### Complications and Reversal of Effect

Hemorrhage is the major complication of LMWH, with some data supporting decreased rates of bleeding compared to UFH. Rates of fatal bleeding are reported in 0–0.8 % and major bleeding in 0–3 % of patients [16]. In the surgical setting, peri-procedural thromboembolic risk assessment, bleeding risk assessment, and physician preference will play a role in determining whether LMWH prophylactic dosing is continued or withheld. For patients receiving therapeutic LMWH dosing, discontinuation should be considered 12–24 h prior to procedure, or longer in patients with renal dysfunction. Therapeutic doses of LMWH should not be restarted for 24 h after a major procedure or after neuraxial anesthesia [19, 32].

In the setting of overdose or life-threatening hemorrhage, protamine is administered IV. Protamine does not fully reverse LMWH but can neutralize the AT effect. Because longer heparin chains bind to protamine, protamine completely reverses the anti-factor IIa activity of LMWH but only reverses 60 % of the anti-factor Xa activity. If immediate reversal is warranted within 8 h of LMWH administration, a protamine dose of 1 mg neutralizes 100 U anti-Xa or 1 mg of LMWH. If bleeding continues, a second dose of 0.5 mg of protamine per 100 U anti-Xa may be administered. Smaller protamine doses are required if the LMWH administration interval is beyond 8 h [34, 35].

HIT and HIT with thrombosis (HITT) are immune-mediated disorders that result from antibodies being formed against the heparin–platelet factor IV complex. The incidence of HITT in critically ill patients ranges from 1 to 5 % and is associated with the development of thrombocytopenia and life-threatening thrombosis in approximately 30–50 % of HIT positive patients [36]. This immune-mediated response typically occurs in patients exposed to UFH or LMWH for 5–7 days, or sooner if the patient was previously exposed. A 50 % decrease in platelet count occurring 4–10 days after the initiation of UFH or LMWH therapy or formation of a new thrombus while anticoagulated may be indicative of HIT. Platelet counts should be measured prior to the initiation of UFH or LMWH and monitored every other day for the first 4–10 days of therapy. The incidence of HIT is approximately one-tenth lower with LMWH than with UFH [37]. In the setting of a HIT allergy or if positive HIT antibodies have been detected, LMWH cannot be used due to cross reactivity between glycosaminoglycans. Direct thrombin inhibitors (DTIs) are the treatment of choice for patients with HIT or HITT [38, 39].

Osteoporosis reportedly occurs less frequently in patients treated with LMWH as compared to UFH, and it typically is associated with long-term therapy [6••].

## Fondaparinux

### Pharmacodynamics and Monitoring

After subcutaneous administration, fondaparinux is rapidly and completely absorbed, exhibiting a half-life of 17–21 h in patients with normal renal function [6••]. Fondaparinux is excreted primarily unchanged in the urine with clearance reduced in patients with renal impairment. Similar to LMWH, with predictable pharmacokinetics, monitoring anti-Xa levels is not recommended during fondaparinux administration.

### Clinical Indication

Fondaparinux has been proven to be at least as safe and effective as treatment of DVT and pulmonary embolism (PE) as LMWH and UFH, respectively [40, 41] (Table 4). Fondaparinux has been studied extensively for thromboprophylaxis in medically ill and surgical patients [42, 43]. In three trials fondaparinux showed superior efficacy in reducing VTE in patients undergoing knee arthroplasty, hip arthroplasty, and hip fracture surgery [44–46]. In a combined analysis, the overall incidence of major bleeding was statistically higher with fondaparinux (2.7 %) compared with LMWH (1.7 %) [47]. However, the incidence of clinically relevant bleeding, as defined as bleeding leading to death, reoperation, or occurring in a critical organ, did not differ between the agents. The differences in efficacy and safety outcomes could be related to dosing as well as the timing of peri-operative drug administration. The administration of fondaparinux given less than 6 h after surgery has been associated with an increased frequency of major bleeding [48]. Holding therapy for at least 6 h post-procedure may be recommended in patients at risk of bleeding. Fondaparinux may be a potential option for thromboprophylaxis in the setting of an HIT allergy but no conclusive data is available [49]. While fondaparinux has been studied in ACS, it has not received Food and Drug Administration (FDA) approval.

**Table 4:** Clinical uses of fondaparinux

Drug	Indications	Dosing, timing, duration	Monitoring	Precautions
Fondaparinux (Arixtra™)	Treatment of VTE [2, 10] Treatment is for 5–9 days; continue treatment until a therapeutic oral anticoagulant effect is established	<50 kg: 5 mg SC daily 50–100 kg: 7.5 mg SC daily >100 mg kg: 10 mg SC daily [2, 10]	CBC Serum creatinine Signs and symptoms of bleeding Anti-Xa level in patients with significant renal impairment, those experiencing bleeding or abnormal coagulation parameters, pregnant patients, obese or low-weight patients, and children Hepatic function	Indwelling epidural catheter Recent spinal or ophthalmologic surgery History of recent major bleed (gastrointestinal, intracranial, etc.) Congenital or acquired bleeding disorders
		Renal impairment CrCl 50–80 mL/min—25 % reduction in total clearance; consider empiric dosage reduction CrCl 30–50 mL/min—40 % reduction in total clearance; consider empiric dosage reduction CrCl less than 30 mL/min—contraindicated		
	Treatment of STEMI and NSTEMI^a^	2.5 mg SC daily		
	Prophylaxis of VTE in major surgery and acute medically ill^a^	2.5 mg SC daily		

*VTE* venous thromboembolism, *SC* subcutaneous, *CrCl* creatinine clearance using the Cockroft–Gault Equation, *CBC* complete blood count, *STEMI* ST-elevation myocardial infarction, *NSTEMI* non-ST-elevation myocardial infarction

^a^Indicates off label use of medication

### Complications and Reversal of Effect

Fondaparinux is contraindicated in patients with severe renal impairment (calculated creatinine clearance less than 30 mL/min). Fondaparinux should not be used for VTE prophylaxis in patients weighing less than 50 kg. Fondaparinux reversal is further complicated by its prolonged half-life [50]. While no specific antidote exists for the fondaparinux-related hemorrhage, recombinant activated factor VII (rFVIIa) administration can normalize coagulation times and thrombin generation [51].

### Direct Thrombin Inhibitors (DTIs)

DTIs exert their antithrombotic effect through direct, selective, and reversible binding to the active site of thrombin. This leads to inhibition of thrombin-catalyzed or -induced reactions, including fibrin formation, activation of coagulant factors V, VIII, XIII, protein C, and platelet aggregation. The hirudin analogs, desirudin and bivalirudin, and argatroban are three currently approved DTIs [52].

### Pharmacology, Pharmacodynamics, and Monitoring

Bivalirudin and desirudin are synthetic analogs of r-hirudin that exert anticoagulant activity by reversible binding at the enzymatic catalytic site and the anion binding site of thrombin. Argatroban, derived from the amino acid arginine, is a small synthetic thrombin inhibitor that reversibly binds non-covalently to thrombin active site [52].

The DTIs differ in their pharmacokinetic parameters (Table 5) [52]. Bivalirudin has the shortest half-life, making it a particularly useful agent in the procedural or peri-procedural period. DTI selection often depends on patient-specific characteristics such as age, compromised cardiac function, hemodynamic instability, and hepatic or renal dysfunction [52, 53]. Critically ill patients typically require lower infusion rates than recommended by the manufacturer due to the presence of comorbidities and organ dysfunction. DTIs are monitored using aPTT, with a goal of 1.5–3 times control or baseline (argatroban), 1.5–2.5 times control (bivalirudin) (Table 6). Desirudin does not need routine coagulation monitoring. The aPTT level should be measured every 6 h until the patient has sustainable therapeutic levels, then the frequency of monitoring can be extended. Because of inconsistencies in aPTT measurements, the plasma diluted thrombin time has shown to be an alternative to for monitoring DTI levels, especially in patients with lupus inhibitors or low levels of vitamin K-dependent factors [54].

**Table 5:** Pharmacokinetic and pharmacodynamic properties of DTIs

Feature	Desirudin	Argatroban	Bivalirudin
Molecular weight (Da)	6963	526	2180
FDA-approved indication	Prophylaxis of DVT in patients undergoing elective hip replacement surgery	Management of HIT, or use in patients with HIT who are undergoing PCI	Use in patients with or at risk for HIT or HITTS who are undergoing PCI
Primary elimination route	Renal	Hepatic	Enzymatic
Elimination half-life	SC = 120 min IV = 60 min	39–51 min	10–24 min
Fraction eliminated unchanged by kidney (%)	40–50	16	20
Laboratory test to monitor	Not required	aPTT, ECT	aPTT, ACT, ECT
Target range	n/a	aPTT: 1.5–3× control	aPTT: 1.5–2.5× control
Effects on INR	Minimal	Moderate to clinically significant	Minimal to moderate

*Da* Dalton, *FDA* Food and Drug Administration, *HIT* heparin-induced thrombocytopenia, *HITTS* HIT with thrombosis syndrome, *PCI* percutaneous coronary intervention, *SC* subcutaneous, *IV* intravenous, *aPTT* activated partial thromboplastin time, *ECT* ecarin clotting time, *ACT* activated clotting time, *INR* international normalized ratio

**Table 6:** Clinical uses of DTIs

Drugs	Indications	Dosing, timing, duration	Monitoring	Precautions
Bivalirudin (Angiomax™)	PCI (with or without glycoprotein IIB/IIIA inhibitor)	0.75 mg/kg IV bolus dose, followed by an infusion of 1.75 mg/kg/h for the duration of the procedure	CBC aPTT ACT PT/INR (false elevation while on infusion) Blood pressure Heart rate ECG Renal function (bivalirudin) Hepatic function (argatroban)	Indwelling epidural catheter Recent major, spinal or ophthalmologic surgery History of recent major bleed (gastrointestinal, intracranial, etc.) Congenital or acquired bleeding disorders Recent cerebrovascular accident Hepatic impairment (argatroban) Renal dysfunction (bivalirudin)
		CrCl less than 30 mL/min, a reduction of initial infusion rate to 1 mg/kg/h should be considered; no bolus dose reduction is necessary		
	Treatment of ACS^a^	Initial IV bolus dose of 0.1 mg/kg, followed by 0.25 mg/kg/h.		
	Treatment and prophylaxis of HITT^a^	0.15–0.2 mg/kg/h, titration to aPTT 1.5–2.5 times control		
Argatroban	Treatment and prophylaxis of HITT	0.5–1.2 μg/kg/min continuous IV infusion to start titration to goal aPTT of 1.5–3 times baseline		
		Begin VKA therapy, measure INR daily. Stop argatroban when INR >4. Repeat INR in 4–6 h, if INR is below desired range then resume argatroban infusion		
	PCI	Bolus: 350 μg/kg		
		Initial infusion: 25 μg/kg/min maintain ACT greater than 300 seconds		
Desirudin	Prophylaxis of DVT in patients undergoing elective hip replacement surgery	15 mg SC every 12 h given 5–15 min prior to surgery but before induction of regional block anesthesia (if used)		

*PCI* percutaneous coronary intervention, *IV* intravenous, *CrCl* creatinine clearance using the Cockroft–Gault Equation, *CBC* complete blood count, *aPTT* activated partial thromboplastin time, *ACT* activated clotting time, *PT* prothrombin time, *INR* international normalized ratio, *ECG* echocardiogram, *HITT* heparin-induced thrombocytopenia and thrombosis, *VKA* vitamin K antagonist, *ACS* acute coronary syndrome

^a^Indicates off label use of medication

### Clinical Indications

DTIs can be used as an alternative anticoagulant to UFH for the treatment of HIT or HIT-T [55]. Argatroban administration significantly reduced the rates of thromboembolic complications in patients with HIT [56]. Bivalirudin has been safely used in critically ill and HIT patients [57]. Argatroban and bivalirudin are indicated as an anticoagulant for thrombosis prevention in patients undergoing percutaneous coronary intervention (PCI). Bivalirudin is indicated for use as an anticoagulant in the treatment of patients with moderate to high-risk ACS, unstable angina/non-ST-segment elevation myocardial infarction who are undergoing early invasive management, and in patients undergoing PCI (Table 6) [5].

### Complications and Reversal of Effect

Hemorrhage is the most common complication with DTIs and no specific reversal agent is available. Anecdotally, rFVIIa has been reported to be useful and could be considered for immediate treatment of life-threatening hemorrhage [58]. DTIs can produce a misleading elevation in the international normalized ratio (INR), complicating the transition to warfarin in HIT. A clinical strategy to bridge safely and effectively should be undertaken in order to avoid thrombosis or bleeding. Steps in a bridging strategy include determining a baseline INR while on the DTI, identifying a target INR level (desired 1.5–2-point increase) while considering the INR elevation induced by the DTI, once INR goal is reached withhold the DTI for 4–8 h, and recheck the INR and aPTT. If the INR is 2–3 with an aPTT close to baseline after the clinician accounts for the independent warfarin-related elevation in the aPTT, the DTI can be discontinued [49].

### Oral Anticoagulants–Vitamin K Antagonists

Vitamin K antagonists (VKAs) produce their anticoagulant effect by inhibiting vitamin K epoxy reductase, which is required for the conversion of vitamin K to its active form vitamin KH2. Vitamin K dependant proteins such as clotting factors II, VII, IX, and X require γ-carboxylation by vitamin KH2 for biological activity [59•].

The relationship between the dose of warfarin and the response varies between patients and is modified by genetic and environmental factors (dietary intake, drug interactions, critical illness, etc.) that can influence the absorption of warfarin, its pharmacokinetics, and its pharmacodynamics [59•, 60•, 61–63].

A wide dosing range is required to maintain a therapeutic INR with relatively low doses often required for the elderly and patients with underlying comorbidities. Nomogram based dosing is considered safer and more effective at reaching targeting INR goals [64]. Larger initial doses suppress proteins C and S, producing a hypercoagulable response and are associated with over-anticoagulation and higher rates of bleeding.

### Clinical Indications

Warfarin is effective for the primary and secondary prevention of VTE, for the prevention of systemic embolism in patients with prosthetic heart valves or AF, for the primary prevention of acute myocardial infarction in high-risk men, and for the prevention of stroke, recurrent infarction, or death in patients with acute myocardial infarction (Table 7) [59•, 65–68].

**Table 7:** Clinical uses of warfarin

Drug	Indications	Dosing, timing, duration	Monitoring	Precautions
Warfarin (Coumadin™, Jantoven™)	Treatment of VTE	Initial dosing: 2.5–10 mg every 24 h (see precautions) titrated to range INR: 2.0–3.0; target of 2.5	Signs and symptoms of bleeding CBC PT/INR	Lower initial dosing (less than 5 mg may be warranted in patients who are debilitated, are malnourished, have congestive heart failure, have liver disease, have had recent major surgery, or are taking medications known to increase sensitivity to warfarin) Cerebrovascular disease Coronary disease CYP2C9 and VKORC1 genetic variation Moderate to severe hypertension Malignancy Renal impairment Recent trauma Malignancy Collagen vascular disease Conditions that increase risk of hemorrhage, necrosis, and/or gangrene, pre-existing Congestive heart failure Sever diabetes Excessive dietary vitamin K Elderly or debilitated patients (lower dosing may be required) Hepatic impairment Hyperthyroidism/hypothyroidism Epidural catheters Infectious diseases or disturbances of intestinal flora, such as sprue or antibiotic therapy Poor nutritional state Protein C deficiency Heparin-induced thrombocytopenia Vitamin K deficiency
	Atrial fibrillation	Initial dosing: 2.5–10 mg every 24 h (see precautions) titrated to range INR: 2.0–3.0; target of 2.5		
	Post-MI	Initial dosing: 2.5–10 mg every 24 h (see precautions) titrated to range INR: 2.0–3.0; target of 2.5		
	Mechanical valve in the atrial position	Initial dosing: 2.5–5 mg every 24 h (see precautions) titrated to range INR: 2.0–3.0; target of 2.5		
	Mechanical valve in the mitral position	Initial dosing: 2.5–5 mg every 24 h (see precautions) titrated to range INR: 2.5–3.5; target of 3.0		
	Mechanical valve in both the atrial and mitral position	Initial dosing: 2.5–5 mg every 24 h (see precautions) titrated to target INR: 2.5–3.5; target of 3.0		
	Bioprosthetic valve in the mitral position	Initial dosing: 2.5–5 mg every 24 h (see precautions) titrated to target INR: 2.0–3.0; target of 2.5 for 3 months		

*VTE* venous thromboembolism, *h* hours, *INR* international normalized ratio, *CBC* complete blood count, *PT* prothrombin time, *MI* myocardial infarction

### Complications and Reversal of Effect

Bleeding is a major concern with warfarin therapy due to the influence of environmental factors and drug interactions in the setting of a narrow therapeutic index. Treatment with VKA increases the risk of major bleeding by 0.3–0.5 % per year and the risk of intracranial hemorrhage by approximately 0.2 % per year compared to controls. The most important risk factors for hemorrhage in VKA therapy include: intensity of anticoagulant effect, time within therapeutic range, and patient characteristics. Higher goal INRs (INR >3) have been directly associated with increased rates of hemorrhage, and patients at high risk of bleeds may benefit from lower target goals [59•, 60•].

Reversal of warfarin’s anticoagulant effect requires withholding therapy. The duration of effect can last up to several days in the absence of reversal agent administration. In patients with clinically significant bleeding, the administration of vitamin K is crucial to reversing the anticoagulant effects of VKAs. In the setting of an INR between 4.5 and 10 and no significant bleeding, the next doses of warfarin should be held and the INR evaluated [60•]. When the INR is >10 and the patient has no significant signs of bleeding, the guidelines recommend holding warfarin and giving oral vitamin K. In the setting of serious/life-threatening bleeding at any INR, warfarin should be held and vitamin K 10 mg by slow IV infusion is recommended. Higher doses of vitamin K are effective but may lead to VKA resistance for more than a week. Vitamin K may be given orally or parenterally, with the IV route providing a more rapid response. The intramuscular or subcutaneous routes are not recommended in the critically ill due to unpredictable absorption. In cases where immediate reversal of the INR is necessary the supplementation of clotting factors with fresh frozen plasma (FFP), or prothrombin complex concentrate (PCC) are more effective. PCCs contain more clotting factors in a smaller volume and have been shown to be more effective in the reversal of warfarin therapy. Recombinant factor VIIa may be of benefit in patients with refractory bleeding in the setting of elevated INRs [60•, 61–69].

Non-hemorrhagic adverse events of warfarin include acute skin necrosis and limb gangrene; these uncommon complications are observed on the third to eighth day of therapy. Skin necrosis is caused by extensive thrombosis of the venules and capillaries within the subcutaneous fat, typically associated with protein C deficiencies. Limb gangrene, however, is due to massive outflow obstruction of the venous circulation of the limb, and can be seen in HIT patients treated with warfarin without adequate initial bridging with a DTI [59•].

Patients on VKAs requiring surgery should hold therapy approximately 5 days prior to the intervention. Depending upon the patient’s history and risk of VTE or arterial thromboembolism, bridging with LMWH or UFH may be warranted. VKA may be resumed 12–24 h post-surgery, depending on bleeding risk and hemostasis. Assessment of the INR should be undertaken before neuraxial anesthesia is performed. For patients with an indwelling catheter who are receiving warfarin, the catheter should be removed when the INR is less than 1.5. Patients with a low risk of bleeding may undergo surgery with an INR of 1.3–1.5 [19–21, 70].

### Target-Specific Oral Anticoagulants

Dabigatran, rivaroxaban, and apixaban are novel oral anticoagulants that offer major advantages over current agents. They have rapid onset and more predictable anticoagulants response that eliminates the need for monitoring. Clinical trials have been completed with all three agents in the prevention and treatment of the three leading causes of cardiovascular death: myocardial infarction, stroke, and VTE. Novel agents have shown reduced or similar rates of thrombosis, major bleeding, and adverse events when weighed against either LMWH or warfarin.

### Pharmacology, Pharmacodynamics, and Monitoring

Dabigatran etexilate mesylate is a prodrug. After oral administration, non-specific plasma and hepatic esterases hydrolyze the compound into the active anticoagulant, dabigatran [71]. Dabigatran is DTI that exerts its action through reversible, competitive binding to the active site on thrombin. Furthermore, dabigatran indirectly exerts an anti-platelet effect by reducing thrombin’s impact on promoting platelet activation and aggregation [72].

Dabigatran is eliminated through renal filtration with up to 80 % of the dose excreted unchanged in urine (Table 8). Dabigatran’s mean terminal elimination half-life is prolonged in patients with severe renal dysfunction. There is no antidote available to reverse or attenuate dabigatran’s anticoagulant effect [72].

**Table 8:** Pharmacokinetic and pharmacodynamic properties of target-specific oral anticoagulants

Features	Dabigatran etexilate	Rivaroxaban	Apixaban
Target	Thrombin	Factor Xa	Factor Xa
Prodrug	Yes	No	No
Dosing	Fixed	Fixed	Fixed
Bioavailability (%)	6	80	90
Food effects	Delay *T*_max_ 2–4 h	Delays *T*_max_	Not reported
Half-life (h)	12–17	5–9	12
Renal excretion (%)	80	65	25
Coagulation monitoring	No	No	No
Antidote	None	None	None
Interactions	P-gp inhibitors^a^	Combined P-gp and CYP3A4 inhibitors^b^	Potent 3CYP3A4 inhibitors^b^

^a^P-glycoprotein (P-gp) inhibitors include verapamil, clarithromycin, and quinidine

^b^Cytochrome (CYP) P450 3A4 inhibitors include but are not limited to: ketoconazole, macrolide antibiotics, and protease inhibitors

Rivaroxaban is an oral, highly selective, direct, competitive inhibitor of factor Xa [73]. Inhibition of factor Xa leads to interruption of the both intrinsic and extrinsic coagulation pathways, thus preventing thrombin generation and subsequent thrombus formation. Rivaroxaban’s inhibition of both free and fibrin-bound factor Xa differentiates its action from LMWH or fondaparinux. Rivaroxaban exerts minimal effect on platelet function.

In patients with CrCl 15–30 mL/min, the dabigatran dose should be reduced to 75 mg twice daily [71]. The manufacturer does not recommend the use of rivaroxaban in patients with an estimated creatinine clearance less than 15 mL/min [74].

### Clinical Indications

While dabigatran has been compared with enoxaparin for VTE prophylaxis, and with warfarin in acute VTE treatment and secondary prevention, it only has FDA approval for stroke prevention in AF (Table 9).

**Table 9:** Clinical uses of target-specific oral anticoagulants

Drugs	Indications	Dosing, timing, duration	Monitoring	Precautions
Dabigatran etexilate (Pradaxa^®^)	Stroke and systemic embolism prophylaxis in non-valvular AF	CrCl >30 mL/min: 150 mg twice daily	No specific assay available	Bioprosthetic heart valves P-gp inducers and inhibitors
		CrCl 15–30 mL/min: 75 mg twice daily		
Rivaroxaban (Xarelto^®^)	Stroke prophylaxis in non-valvular AF	CrCl >50 mL/min: 20 mg once daily with the evening meal	No specific assay available	Spinal/epidural anesthesia or puncture CrCl <15 mL/min in non-valvular AF CrCl <30 mL/min in treatment or prevention of DVT, PE P-gp inducers or inhibitors CYP3A4 inducers or inhibitors Pregnancy
		CrCl 15–50 mL/min: 15 mg once daily with the evening meal		
	Treatment of DVT or PE	15 mg twice daily with food for 21 days then 20 mg daily with food for remaining treatment		
	DVT or PE secondary prophylaxis	20 mg once daily with food		
	DVT prophylaxis following hip or knee replacement surgery	10 mg once daily for 35 days (hip replacement) or 12 days (knee replacement)		
Apixaban (Eliqiuis^®^)	Stroke and systemic embolism prophylaxis in non-valvular AF	5 mg twice daily or 2.5 mg twice daily in patients with at least two of: age >80 years, body weight <60 kg, serum creatinine <1.5 mg/dL	No specific assay available	

*CrCl* creatinine clearance, *DVT* deep vein thrombosis, *PE* pulmonary embolism

RE-LY was a non-inferiority trial designed to determine the long-term safety and efficacy of dabigatran administered twice daily as compared to warfarin (INR goal 2.0–3.0) in patients with non-valvular AF [75]. Patients were required to have at least one addition thromboembolism risk factor. The primary efficacy outcome was defined as the occurrence of stroke or systemic embolism. The dabigatran 150 mg twice daily regimen was statistically superior to warfarin in reducing the rate of stroke and systemic embolism, 1.11 % per year versus 1.69 % per year, respectively (*p* < 0.001). As any other anticoagulant, bleeding is the major adverse event. In Re-LY trial, the primary safety outcome was major bleeding. There was no difference in the rate of major bleeding in the dabigatran 150 mg group compared with the warfarin group.

Rivaroxaban has been studied in a large clinical trial program and has FDA approval for a variety of indications. The orthopedic surgery program compared rivaroxaban to enoxaparin for VTE prevention in patients undergoing total hip and total knee arthroplasty [76–79]. The primary efficacy endpoint was total VTE, the composite of any DVT, non-fatal PE, and all-cause mortality. In the RECORD1, -2, -3 and -4 studies rivaroxaban 10 mg daily was superior to enoxaparin and associated with a similar safety profile. Rivaroxaban has been evaluated for stroke prevention in patients with non-valvular AF [80]. In the ROCKET-AF trial rivaroxaban 20 mg once daily was non-inferior to warfarin in reducing all-cause stroke and non-central nervous system embolism in with a similar rate of major bleeding. Rivaroxaban has been studied for the acute DVT and PE treatment and for the long-term secondary prevention of recurrent VTE [81, 82]. The EINSTEIN-DVT study found rivaroxaban 15 mg twice daily for 3 weeks followed by 20 mg daily was non-inferior to enoxaparin followed by a VKA in the prevention or recurrent VTE [81]. In the EINSTEIN-Extension trial, rivaroxaban 20 mg daily extended for an additional 6–12 months significantly reduced recurrent VTE without an increase in major or clinically relevant non-major bleeding when compared with placebo. In EINSTEIN-PE rivaroxaban was found to be non-inferior to enoxaparin combined with a VKA in the prevention of recurrent VTE while providing a significant reduction in major bleeding [82].

While apixaban has been evaluated in thromboprophylaxis following orthopedic surgery, secondary prevention of VTE, and ACSs, its sole FDA approval is in stroke prevention in AF [83•].

In the ARISTOTLE trial, investigators compared apixaban 5 mg twice daily with warfarin titrated to a goal INR of 2–3 [84]. The primary outcome, a composite of stroke and systemic embolism, occurred significantly less frequently in the apixaban patients compared to the warfarin patients. Bleeding was significantly less frequent in the apixaban patients compared to the warfarin patients across several bleeding definitions tested.

### Complications and Reversal of Effect

The most common adverse events reported with dabigatran include dyspepsia, dizziness, headache, dyspnea, and shortness of breath. Abdominal pain and gastritis-like symptoms may be related to the capsule formulation which can be combated by taking the medication with food [83•].

There are no specific coagulation assays for laboratory monitoring of novel oral anticoagulants. Thrombin time and aPTT can be used to detect the presence of dabigatran in the plasma [85]. Similarly, rivaroxaban and apixaban prolong prothrombin time and aPTT. The role of other newer assays (Heptest, prothrombinase-induced clotting time, Anti-FXa chromogenic assays) have yet to be established.

Currently, there is no antidote available to reverse dabigatran, rivaroxaban, or apixaban. In the event of overdose, the early use of activated charcoal is recommended [83•]. Cessation of dabigatran, rivaroxaban, or apixaban therapy may be sufficient to reverse any excessive anticoagulant effect due to their short half-lives. While dialysis, for 2–3 h removes up 60 % of dabigatran, it has no impact on rivaroxaban or apixaban [85].

Limited data exists for treatment of life-threatening bleeding produced by novel anticoagulants. Recent trials have suggested PCCs or rFVIIa may offer benefit by normalizing coagulation parameters [74, 86•, 87]. Their evaluation, use, and role in the clinical setting are still required. In the event of bleeding symptomatic treatment of the hemorrhage should be initiated [83•].

For the novel agents, confusion and debate may ensue if prescribed in patient populations, such as those with pregnancy, mechanical heart valves, and thrombophilias, where little study data and clinical experience exists. Similarly, surgical and invasive procedures add additional levels of complexity where therapy may be continued, interrupted, or replaced with short-term parenteral or ‘bridge’ therapy.

## Conclusions

UFH, LMWHs, fondaparinux, DTIs, and warfarin have been studied and employed extensively for prevention and treatment of thrombosis. Novel oral anticoagulants have emerged from clinical development and are expected to replace older agents with their ease of use and more favorable pharmacodynamic profiles. Hemorrhage is the main concerning adverse event with all anticoagulants. With their ubiquitous use, it becomes important for clinicians to have a sound understanding of anticoagulant pharmacology, dosing, monitoring, and toxicity. Working knowledge becomes crucial for intercepting and averting problems.
